# The Hospital. Nursing Section

**Published:** 1902-01-25

**Authors:** 


### The Hospital.

### Nursing Section.

Contributions for this Section of "The Hospital" should be addressed to the Editor, "The Hospital" Nursing Section, 28 & 29 Southampton Street, Strand, London, W.C.

No. 800.—Vol. XXXI. *SATURDAY, JANUARY 25, 1902.*

### Notes on News from the Nursing World.

### QUEEN ALEXANDRA AND WOLVERHAMPTON DISTRICT NURSES.

Her Majesty the Queen has written to Miss Lowe, Honorary Secretary of the Queen Victoria Nursing Institution at Wolverhampton, graciously consenting to become a patroness of the institution.

### A CORONATION MEMORIAL.

It has been decided by the governors of the North Staffordshire Infirmary, Stoke-on-Trent, to commemorate the Coronation year by the erection of a new block of buildings to be called "King Edward VII. Home for Nurses," adjoining the main building and connected with it by a covered way, at a cost of about £10,000. Accommodation will be provided for about 50 nurses. The fact that the foundation-stone of the Infirmary was laid in 1866 by the King and Queen, then Prince and Princess of Wales, suggested the idea of the memorial, which, we are sure, will commend itself to the Sovereign. We hope that the Coronation may be commemorated in a similar manner in many other places.

### THE WAR NURSES.

The *Dunera* arrived at Southampton from South Africa on Tuesday, the following nursing sisters forming the "ship's staff":—A.N.S. Acting Superintendent M. G. Hill, who rejoins the ship; I. Creighton, M. Marius, M. Meade, M. Herring, all A.N.S.R., and rejoining the ship; and N. D. Redstone, New Zealand A.N.S.R., who wishes to resign, and is desirous of returning to India. M. Stephenson and C. M. Mello, both A.N.S.R., were invalided home. The *Canada* arrived on the same day, with M. D. Knapp, A.N.S.R., who requires one month's leave and returns to S. Africa; E. C. Blakestone, civil nurse, who is not desirous of returning to S. Africa; and A. M. Clarke, who desires three months' leave and returns to S. Africa.

### MR. LONG'S COMMITTEE OF INQUIRY.

We heartily congratulate the President of the Local Government Board upon his action with regard to the nursing of the sick poor in workhouse infirmaries. Only last week we expressed the conviction that the desire of the Board, under the present *régime*, is to level up the nursing arrangements to present-day needs, and the appointment, by Mr. Walter Long, of a committee of inquiry strongly confirms this view. We have reason to believe that the constant insistence of The Hospital upon the urgent importance of a reform of the system, or want of system, prevalent in so many workhouse infirmaries, has had a considerable influence in deciding the Local Government Board to make an exhaustive investigation. The departmental committee—which consists of Mr. Grant Lawson, M.P., parliamentary secretary, Mr. W. E. Knollys, C.B., the well-known chief general inspector, and one of the assistant secretaries, and Dr. A. H. Downes and Dr. A. Fuller, Poor Law medical inspectors, Mr. R. H. A. G. Duff acting as secretary—will inquire and report as to the qualifications of nurses and probationers, the difficulties in obtaining an adequate supply of these officers, and the regulations necessary to define the respective duties of the master or matron and of the superintendent nurse. With regard to the second of these points, a glance at our file for last year will prove the reality of the difficulties in obtaining nurses at a certain class of workhouse infirmaries. But while, to take the most recent cases, the Atcham Board of Guardians have changed their superintendent nurse seven times in two years, and the Bristol Board have, or had, five vacancies for nurses unfilled, such institutions as Southwark and Birmingham Infirmaries can always command the services of as many nurses as they require. If Mr. Long's Committee will carefully ascertain the reasons for this striking contrast they will soon be in possession of the key to the situation. We understand that they will commence to sit early next week, and that the proceedings will be private. We are also officially informed that any person desirous of giving evidence before the committee should communicate with the secretary at the offices of the Local Government Board, Whitehall, S.W., informing him of the particular points to which their evidence would be directed, and of any special qualifications they may possess for giving such evidence. Some of our readers, and especially some of our well-informed correspondents, will, no doubt, be glad to avail themselves of the opportunity thus afforded.

### THE LATE MATRON OF THE WELSH HOSPITAL.

Some interesting details respecting the funeral of the late Miss Lloyd, who was in charge of the Welsh Hospital for nearly two years, reach us from one of the sisters in Pretoria, who knew her well. The order for dress was not strictly given, but most of the nurses were attired in bonnets and cloaks. The nurses of No. 7 were all of one mind, and wore cotton dresses, red capes, sailor hats: they stood together on one side of the grave. The gun-carriage started from the sisters' hospital at about 5 p.m., with an escort. The bearer company consisted of the N.C.O.'s of No. 2 Hospital, and there were two men riding ahead. It was more than two miles to the cemetery, and the procession walked the whole way. No. 7 Hospital sisters met the coffin at the grave. As the gun-carriage came up to the cemetery it was met by the bagpipes and muffled drums of the Gordons, and the band struck up the "Dead March" as the coffin was carried to the grave. A very short service was conducted by the chaplain, and the coffin was almost at once lowered into the grave by four sergeants of the

R.A.M.C. The pipers then stood over the grave, and when the service finished played the "Retreat." Finally the band played "Peace, perfect peace." There were numerous wreaths, but very poor flowers, owing to the season. All the R.A.M.C. officers in town attended. General and Lady Maxwell, some other generals, and a number of staff officers were present. The grave is a short distance from Prince Christian Victor's.

### BERKSHIRE HOSPITAL AND THE ROYAL NATIONAL PENSION FUND.

In the course of an interview with our Commissioner, which appears in another part of the paper, the matron of the Royal Berkshire Hospital at Reading mentioned, as an important inducement offered to the staff of the private nurses' institution, the fact that the governors make a point of helping the nurses to help themselves. It is one of the regulations of the institution that when a nurse has been in the employment of the hospital for six months she will be expected, if she has not already done so, to take out a policy for a pension, to commence at the age of 50 or 55, in the Royal National Pension Fund for Nurses, the amount of annual premium to be paid being at least £7. While the nurse is thus called upon to do her share in providing for the future, the hospital does its part by taking out, at the same time, on her behalf, a policy of equal value, providing that the premium on each policy does not exceed £7 a year. At the expiration of five years the benefit of the policy effected by the hospital on behalf of the nurse is considered as belonging to her, with the understanding that it is to be assigned to her when her pension falls due, or otherwise when she leaves the hospital. Of course, it is required that she shall comply with the rules, conduct herself properly, and pay the premiums on her own policy while she remains in the service of the hospital. But these reasonable and necessary conditions being fulfilled, the nurses attached to the institution make sure, at the end of five years, of provision for themselves in the future. All the sisters are members of the Royal National Pension Fund under similar conditions. We are not surprised that with this and other encouraging features of service the governors find the private nursing branch rapidly gaining popularity. With respect to the Pension Fund, members already knew that 1901 was a record year in its existence. But we may mention that more nurses joined the fund, the new policies issued being over 830; more money was received in premiums, the net amount being upwards of £80,000; more was paid out in pensions, the sum exceeding £6,000; and the sick pay distributed to policy-holders was over £1,600, or more than during any previous year since the Fund was established.

### THE NURSES' HOME AT GUY'S.

In his speech at the Mansion House meeting, reported last week, Mr. Cosmo Bonsor alluded to the reason which led the governors of Guy's Hospital to embark in the erection of the nurses' home, which is rapidly approaching completion. The treasurer told his audience how one January morning in 1898 the late Mr. Henry Raphael called at the hospital, and having visited the attics in which the nurses were lodged, and inspected an adjoining site where it was possible to build a nurses' home, gave a cheque for £20,000 towards it. Would that there were many men of this stamp about! As our readers know, the greatest of all the gains of the new home will be that each nurse will have a room to herself. But the building is altogether on a large scale; and though as a training school Guy's already stands very high, many improvements in the details of management will be possible when the matron can gather her forces under the roof of the new home.

### COMPLETION OF THE NURSES' INSTITUTE AT CAPETOWN.

A very interesting ceremony took place last month in connection with the Victoria Nurses' Institute at Capetown, which was commenced in the year of the Diamond Jubilee. The building was started free of debt, but it was soon found that if the original design and intent were to be fulfilled more accommodation must be provided. The money for this extension has been obtained by placing a mortgage on the property in Hof Street, near the Mount Nelson Hotel, and the enlarged institute was opened by the Governor, in the presence of a large and representative gathering. The front portion has been entirely rebuilt, and a second floor added, while the wing has been renovated. On the ground floor are matron's and nurses' sitting, dining, and recreation rooms, as well as offices, and above are the bedrooms. The appointments are homelike and comfortable without being extravagant, and there are rooms for a matron and twenty-nine nurses, besides the domestic staff. All the nurses employed are fully trained. The institute is self-supporting, each nurse paying for board and lodging, and returning to the matron a trifling percentage of the fees she makes. His Excellency in his speech on the occasion alluded to the desire that the institute might be able at some time to extend its operations and nurse the sick poor in their own homes; and for that reason he had declined to help in the endowment fund for Queen Victoria's Nurses now being collected in England. Lady Buchanan subsequently said that she had from the first tried to work a scheme of district nursing from the institute, but it was full of difficulty, the mixed races being a serious trouble. By degrees she hoped, however, to educate people up to the district nurse, and meanwhile they could at least do good work with maternity nurses, who were increasingly needed. She paid a tribute to the two nurses who had lost their lives in discharge of their duty during the year—Nurse Keyser who died of plague, and Nurse Webster who took typhoid whilst she was nursing soldiers at Kroonstad—concluding with an acknowledgment of the good work done for the institute by the present matron, Miss Damon.

### HOSPITAL NURSES AT FIRE DRILL.

The nurses at the Royal Alexandra Hospital for Sick Children, Brighton, have just undergone fire drill. After the fire appliances of the hospital had been tested, the nurses were put through a course of drills by the superintendent of the Brighton Borough Police Fire Brigade, including the use of the appliances with water turned on from the hydrant inside the building. They were also tried with the fire-escape canvas sheets, all the nurses going down the sheets from the first and top floors, as well as several of the patients. The operations were carried out in the presence of the resident medical officer and matron.

### WHEN IS A NURSE NOT A NURSE?

A VERY curious case has been heard in the Chancery Court of Lancashire. The plaintiff, Miss F. M. King, proprietress of a nursing institute at Southport, sought for an injunction to restrain Miss Selina Wilson, a nurse, from undertaking nursing work in Southport, or within a radius of 25 miles, for the residue of a term of two years from August 1900. That Miss Wilson entered into a contract with Miss King under which she agreed not to nurse in the district was not denied; but the point of dispute was whether she had violated the contract by nursing a lady at Southport. It appears that after she quitted Southport, Miss Wilson was employed at the West Kent Central Hospital in Maidstone, and there nursed Mrs. Wylie. Subsequently she left the West Kent Hospital, and, it was alleged, is now nursing a lady on her own account at Southport. The defence was that at the time Miss Wilson severed her connection with the West Kent Hospital, she accepted the post of companion to Mrs. Wylie. Evidence on her behalf went to show that she acted as such, rather than as nurse; but Miss King contended that she fulfilled the duties of a nurse, and that this injured the institute at Southport. We are not surprised that the Vice-Chancellor postponed his decision, which involved a solution of the rather complex problem, When is a nurse not a nurse? for nearly a week. On Tuesday, however, judgment was given in favour of the plaintiff, the Vice-Chancellor being of opinion that the defendant could not divest herself of her professional character, though acting as companion, for while she no doubt performed other duties, she was also available as a nurse if her services were required.

### THE BALLOT AT BARRY.

FOLLOWING a seasonable entertainment to the poor, the Barry District Nursing Association held its annual meeting. The chairman was able to congratulate the company upon a sound financial position, but the treasurer said that he should like to see the whole of the debt now remaining on the institution wiped out at an early date. One new departure was agreed to, namely, the election of a portion of the members of the executive committee by ballot. The remainder are elected by the Friendly Societies, Trades Council, District Council, and the medical men of the town. In this way a representative body is secured. The object of departing from the plan of electing half the members by open vote seems to be to do away with the necessity for an annual meeting. A general meeting may, however, be held at any time within a month of the date of a requisition sent to the secretary signed by ten subscribers. The advantages of the change are not apparent.

### THE RICHMOND CASE.

THE people of Richmond, Yorkshire, must be glad that the woman who described herself as superintendent of the Richmond Nurses' Home was brought to book by the National Society for the Prevention of Cruelty to Children, and that in consequence of her conviction a young child who was an inmate of the "home" has been sent to the workhouse. The woman's cruelty might, possibly, have been made known before, if a nurse in the home had not been too timid to speak out. This nurse, who said at the trial that she heard the complainant scream several times, and saw her face cut and bleeding, confessed that she had known Boultbee to ill-treat other maids, but "she had kept it to herself." A nurse who sees her fellow-creatures, incapable of adequately defending themselves, ill-treated, and yet maintains silence, hardly grasps the obligations of her vocation.

### A PROMISING START AT MORETONHAMPSTEAD.

THE cottage hospital established at Moretonhampstead, and generously endowed by the Hon. W. F. D. Smith, M.P., and Lady Esther Smith, has already justified its existence. It was opened last March for the reception of patients, and has since been carried on most efficiently by the matron-nurse. The patients in number considerably exceeded expectation, and the nursing staff has therefore had to be strengthened. We are glad to hear that arrangements have been made for the junior nurses to have the benefit of lectures on medical and surgical nursing.

### THE JUNIUS S. MORGAN BENEVOLENT FUND.

THE donation of £1 from "A Hospital Sister," acknowledged by the Secretary of the Royal National Fund for Nurses last week, was a contribution to the Junius S. Morgan Benevolent Fund.

### DISTRICT NURSING AT RYDE.

IN a paragraph last week respecting the opening of a Nursing Institute at Ryde the work of a most deserving institution was inadvertently overlooked. The sick poor of the town are already provided for by the Ryde District Nursing Association, which was founded in 1887 for their benefit, and was in 1893 affiliated to Queen Victoria's Jubilee Institute. We are informed that at present the staff at the Home in Monkton Street, Ryde, consists of four nurses, three for general and one for maternity cases. It has just been enlarged and improved in memory of the late Dr. Alexander Davey, to whose unfailing interest and support the present satisfactory condition of the Association is so largely due.

### CROYDON INFIRMARY TROUBLES.

AT the last meeting of the Croydon Board of Guardians the business transacted in private included the consideration of a letter from the Local Government Board respecting the administration of the Workhouse Infirmary. Generally speaking, the difficulties between the matron and the Guardians have been discussed in public, and it is curious that this letter should have been taken as private business. The only authentic information available is that the Local Government Board have declined to interfere in the dispute at present.

### SHORT ITEMS.

AN entertainment on behalf of the new Nursing Home at Charing Cross Hospital will be given in a ballroom of the Savoy Hotel on the afternoon and evening of February 10th and 11th. There will be many attractions.—Mr. Laurie Phillips was responsible for the entertainment which was given to the patients of the Cancer Hospital on Thursday evening last. The programme was well rendered by the various artistes. The usual vote of thanks and the National Anthem closed a very enjoyable evening.

### Lectures on Gynæcology for Nurses.

By Robert Jardine, M.D., M.R.C.S., F.F.P. and S.G., F.R.S.E., Senior Physician to the Glasgow Maternity Hospital, Examiner in Midwifery to the University of Glasgow.

### LECTURE II.—SOME INSTRUMENTS USED IN ORDINARY GYNÆCOLOGICAL EXAMINATIONS.

In this lecture we shall consider the commoner instruments used in gynæcological work. They should all be made of material which can be thoroughly sterilised, preferably by boiling. Metal, glass, vulcanite, celluloid, and rubber are the chief materials from which they are made. The two first can be boiled, but the others will have to be sterilised by soaking in an antiseptic solution. A nurse must bear in mind that all the instruments under her care must be kept scrupulously clean, and that they must be rendered perfectly aseptic before being used.

*Specula.*—These are instruments designed for the purpose of enabling one to examine the vaginal canal and cervix by sight, and also to do any operations on these parts. They are tubular, bivalve, duck-billed, or spatular in form.

*Tubular.*—Fergusson's speculum is a tube with the inner end slanting or bevelled, and the outer end somewhat like the mouth of a trumpet. It is usually made of vulcanite lined with glass to reflect the light. Some are made entirely of glass and others of metal. They are used for examining the cervix, and especially for making applications to it and the interior of the uterus. They are of various sizes.

*Method of Using.*—The patient may either lie on her left side, or on her back, with the limbs well drawn up. In all examinations the external genitals should first be carefully cleansed with a lysol solution. Your own hands should be aseptic and warm. Take the speculum in your right hand, grasping it by the outer end, separate the labia with your left fingers and insert the point of it into the vulva, pressing it well back against the perineum. Press it firmly but gently backwards and upwards, and give it a half turn so as to catch the cervix. The pointed end should be in the posterior fornix, and the cervix should be seen projecting into the bevelled end of the speculum. To make the instrument slip in easily the outer surface may be smeared with antiseptic vaseline, but if it has been soaking in lysol solution this is not necessary. A speculum of this kind should not be used in examining cases of cancer of the cervix, as serious bleeding may be caused. Neither should it be used during menstruation, when there is much inflammation, or in unmarried girls as a rule. In these latter cases no form of specula should be used unless there is very good reasons for the examination.

*Bivalve Specula.*—There are various forms of these: Reid's is about the most convenient. It consists of a long posterior blade and a shorter anterior one, so hinged that it can be widely opened at the inner end when it is in the vagina. It is passed closed with the blades lying on either side of the vagina, and is then rotated so as to bring the longer blade against the posterior vaginal wall, and the shorter one against the anterior wall. It is then opened, and the cervix can easily be seen. It can be used with the patient either on her back or left side. The lateral walls of the vagina can be seen. In some forms of the bivalve specula the anterior blade is not solid, but formed of two prongs so that the anterior vaginal wall is exposed to view between the prongs. Before withdrawing this speculum, care must be taken to close it. I have known a careless student withdraw it open.

*Duck-billed Specula.*—Marion Sims invented this form. There are many different forms now in use, but they are all on the same principle as Sims' original one. The original one consisted of a larger and smaller blade shaped somewhat like a duck's bill, and joined by a slightly curved handle. It is really a vaginal retractor. Only one blade is used at a time. The larger blade is inserted and the perineum and posterior vaginal wall drawn back. The instrument is kept in position by grasping the smaller blade. The patient may be in what is known as the Sims' position, *i.e.*, on her left side with her left arm behind her and the limbs well drawn up, or she may be in the lithotomy position, *i.e.*, on her back with the thighs apart and well bent, and her hips at the edge of the bed or table. If the anterior vaginal wall is very lax it may be necessary to keep it out of the way by means of another speculum or a retractor, or the cervix may be caught and drawn down by means of a sharp hook or forceps. For operations in the vagina, cervix or uterus this is the best form of speculum. The smaller blade may be used when the vagina is narrow, and it is sometimes used in examining the rectum. Sets of movable blades are sometimes used, and sometimes a weight is attached to the handle so as to keep it in position without holding it. Cervical specula are occasionally used for examining the cervical canal, but they are of very little use.

*Rectal Specula.*—The smaller end of Sims' vaginal speculum may be used, or a small bi-valved one; but the ordinary rectal speculum is tubular, closed at the end, and with a wide slit up its side. It is lined with glass to reflect light. By turning it round the whole lower part of the rectal wall can be examined. Vesical specula are used for examining the bladder. The urethra will usually have to be dilated first. A small electric lamp is usually attached to illuminate the bladder. It is then known as a cystoscope. For ordinary vaginal work daylight is the best illuminant, but when it cannot be got an electric light or an ordinary oil lamp with a reflector will do.

*Uterine Sound.*—It is usually known as Simpson's sound. It is made of a metal which can be easily bent. At $2\frac{1}{2}$ inches there is a knob on it which indicates the usual length of the uterine cavity and it is marked off in inches and parts of inches. It may be used to ascertain the length of the uterine cavity, whether or not the canal is patent, the direction of the uterine axis, the condition of the interior of the uterus, to differentiate between a polypus and an inverted uterus, etc., and it is sometimes used to replace a displaced uterus. Normally, the passage of it should not cause pain or bleeding, but in inflammation of the endometrium it will cause both. A sound is an instrument which a nurse should never pass. It should not be passed during menstruation, or when there is much inflammation; and before passing it the doctor should make sure the woman is not pregnant. He should also, if possible, know the direction of the uterine axis.

*Playfair's Probes.*—These are probes which are used for making applications to the interior of the uterus. A thin film of absorbent cotton wool is placed in the palm of the hand and the dampened probe is placed at one edge of the wool and then rolled as it were over the wool. The wool should cling closely and smoothly to the probe for about $2\frac{1}{2}$ inches. The dressed probe may be used for swabbing out the uterus or for applying substances such as pure carbolic acid or iodised phenol to the endometrium.

*Volsella Forceps.*—These are long catch forceps which are used for steadying the cervix or drawing it down. A tenaculum or long sharp hook is sometimes used for the same purpose. There are many other instruments for gynæcological purposes, but the ones I have mentioned are those which are ordinarily used for examining a patient. The others we shall speak of when dealing with the different operations.

### The Nurses of the Royal Berkshire Hospital.

A CHAT WITH THE MATRON (BY OUR COMMISSIONER).

Important changes have been made during recent years in the accommodation provided for the nursing staff of the Royal Berkshire Hospital, but before I asked the matron to tell me about them on the occasion of my visit to Reading the other day, I availed myself of the opportunity offered by Miss Easton to go through some of the wards, to inspect the nurses' home, and to have a look at the new quarters of the private nurses. At the present moment considerable improvements in the hospital itself are on the eve of initiation, and when these, which will include the erection of a large new kitchen and the introduction of new machinery in the laundry, have been effected, it is probable that the Governors will decide upon additional alterations. In that case, there is a prospect that the one weak point yet remaining in connection with the housing of the nurses will be remedied. Meanwhile, the home for those engaged in the hospital is very conveniently arranged, and very comfortably fitted up, while the interior of the handsome house in which the private nurses reside admirably corresponds to the exterior, and is quite a model of its kind. If there has been no extravagance in furnishing, every possible pains seem to have been taken to render it attractive to the inmates, who have also the advantage of being able to enjoy the air in a charming garden at the back.

"Do many of your probationers join the private staff when they have finished their training?" I asked when we had concluded the tour of inspection.

"Most of them are keen to do so," replied the matron, "and so far we have always had vacancies in the private nurses' institution for all who we have considered suitable and who desired to join. I am pleased to say that three of our nurses from the hospital staff have been out in South Africa during the war, as well as three from the private staff. Two have returned, one is now on her way home, and three are still away."

"What is the number of probationers now undergoing training?"

"Thirty-six in different stages. We call them senior and junior nurses. There is an assistant matron, whose services I immensely value, and there are seven sisters and one night superintendent. The private nurses number thirty-six and a home sister."

"To take the hospital first. What are the hours of duty for the day nurses?"

"They breakfast at 6.30, and come into the wards at 7. They have half an hour for lunch. One detachment dine at 1.15 to 1.45, and the other at 2 to 2.30. Tea is from 5 to 5.30 or 5.30 to 6; and supper from 8 to 8.30 or 8.30 to 9. Prayers are said in the chapel at 9, after which the nurses go to their sitting-room or bedroom, as they please. Lights must be out at 10. Two hours off duty are allowed daily, but I should like to give three. I hope to be able to arrange the extra hour. They have a day off once a month.

### Hours and Diet of Night Nurses.

"How long are the night nurses on duty?"

"They are expected to be in the wards after prayers, and they are on duty until 9 A.M."

"Is their diet the same as that of the day nurses?"

"Yes. It is difficult to give them much change during the night, but we vary it with cold ham, bacon and eggs, and sometimes sardines or potted meat. They dine at 9.15 A.M., and the dinner is in no way inferior to that provided for the day nurses. An additional nurse is on duty in the night and can relieve the night nurses when they have their meal."

"What is the number of night nurses?"

"Eight, and there is also a night superintendent. This means practically one to each ward, as there are two wards with only ten beds in each of them. Four of the wards contain 28 patients each, and the two children's wards 14 each. In all there are 160 beds."

"Do you get any complaints from the night nurses about food or hours?"

"No. I think that they are quite as happy as the day nurses. They all sleep at a house, rented for the purpose, close to the Private Nurses' Institution. The night superintendent is in charge, and one of the porters and his wife act as caretakers. After dinner the night nurses go out, and bedtime is either from 10.15 A.M. to 5.15 P.M., or from 12.30 P.M. to 7.45 P.M. As a rule they alternate the hours; and the variety is much appreciated. If any extra cases need attention, we put on an extra nurse in the night."

"From the private staff?"

"Now and then. But I never put the private nurses on in the hospital unless a patient wants a special nurse. The private nurses are not expected to do ward work."

"How long are the night nurses off duty?"

"From 10 A.M. to 12.30 P.M., or from 6 P.M. to 8.30 P.M. When they rise at 5.15, they may either have tea at the day-nurses' table in the hospital, or make tea for themselves in their rooms in Portland Place; when they rise late they make tea before they go to bed."

"Is three months the period your nurses remain on night duty?"

"Generally, it is. I think it is better not to keep them on longer. They have two nights off duty in the middle of the three months."

### The Improvements.

Do all of the nurses sleep outside the hospital building?"

"With the exception of eight probationers. As you have seen, we have still a few cubicles, all however with a window, and two with a fireplace. Each nurse has a cubicle to herself, and they are perfectly healthy. But they are only retained because of the exigencies of space."

"When were the improvements made?"

"We began in the early part of last year. The first thing to be done was to move the private nurses from the home to 24 Portland Place, so that the former might be available for the hospital nurses. Up to that date the only sitting-room accommodation the hospital nurses possessed was the general dining-room, and the corner adjoining which is now used as a tea-room for the senior nurses. Moreover, each of the sisters slept in a room at the end of the ward, and had to go through it whenever they went out in their ordinary attire. Now the hospital nurses are entirely separated from the private nurses, and there is one sitting-room for the sisters, a second for the senior nurses, and a third for the juniors with a grand piano. All but two who sleep in the home have a bedroom to themselves. For these two there is one room divided like a cubicle."

"Who is responsible for order?"

"The senior sister. On fine days the nurses often walk across to the hospital, but there is a subway for use at night and in case of bad weather. The nurses have a tennis court of their own, and most of them are cyclists."

### The Salaries.

"The training is for three years?"

"Yes. We have no paying probationers; but there is an entrance fee of two guineas, which is intended amongst other reasons to keep out persons who only want to enter for a whim. It answers its purpose and has not been objected to. We receive probationers from the age of 23 to that of 33, and they all come for a month on trial, the month being included in the three years."

"What are the salaries?"

"The salary of the nurses is £8 the first year, £15 the second, and £20 the third. If they stay on in the hospital beyond the three years we pay £24 to £26. Last year the Board raised the salaries of the sisters, which now commence at £35, and rise annually to £45. The whole of the staff have three weeks' holiday each during the year."

### Workhouse Infirmary Nurses at Lectures.

"Is there anything exceptional about the instruction?"

"Four members of the medical staff deliver courses of lectures on anatomy, physiology, medical and surgical ursing, and I also lecture on nursing and general administration. At the end of each series of lectures an examination is held. One of the medical staff is also the medical officer to the Reading Board of Guardians, and eight of the workhouse infirmary nurses attended all the lectures in order to go in for the examination."

"Of course they do not get the hospital certificate?"

"No; but if they pass our examination the Local Government Board consider them qualified for superintendent nurses. We have our certificates neatly engraved on parchment, and presented to the recipients in cases."

"What about uniform?"

"Indoor uniform is provided; but I do not encourage the wearing of outdoor uniform so far as the hospital nurses are concerned. Although we have a chapel, we are undenomi national.

### The Private Nurses' Institution.

"When the new home of the Private Nurses' Institution was opened last year, how large was the staff?"

"It had dwindled down in December, 1900, to sixteen, and we were unable to get any more by advertising. The reason no doubt was because we only offered a salary of £30, rising to £34. Sixteen were nothing like adequate to meet the demand, and, as we so often had to refuse to supply nurses, it was determined to reorganise the staff and put them as far as possible on the same basis as the private staffs attached to London hospitals. The salary was raised to £35 at the start, increasing £5 annually to £45. A bonus of 5 per cent. is also given them on their earnings."

"And you have no difficulty now in obtaining a sufficient supply?"

"None whatever. But we had to increase our fees from a guinea and a-half to two guineas a week. I am sorry for those people who can ill afford to pay more, but there has been no falling off in the number of applications for nurses. Last year there were 398 applications, and we sent out nurses to 352 cases. We had to refuse cases in the early months, but since we have had thirty-six nurses we have been able to undertake nearly all."

"You, as matron, have charge of the private nurses?"

"Yes, and all the business of the institution is done here. One of our former nurses acts as home sister and housekeeper. The staff are given indoor and outdoor uniform, including summer and winter cloaks. Quarantine quarters are provided, consisting of a disinfecting room, a sitting-room, kitchen, three bedrooms, and a bathroom. Every nurse who attends an infectious case goes there for four days. Typhoid fever is included. For scarlet fever the period of quarantine is extended to a week. The nurses often attend the sick poor, and people pay for them."

### The Pension Fund.

"The Royal Berkshire Hospital, I know, works in connection with the Royal National Pension Fund for Nurses. Are all the private nurses members?"

"It is one of the regulations that they should join. All the sisters in the hospital belong to the Fund, and the Board pay £7 a year each for them. It is not compulsory for probationers, but as soon as they finish their training and remain in the employment of the Hospital they take out a policy, paying at least a premium of £7. The hospital also takes out one for them for £7. After a nurse has been in the service of the hospital for five years the benefit of the policy effected by the hospital on her behalf belongs to her, and is assigned to her when the pension falls due, or otherwise upon leaving the hospital, if she has complied with the rules, has conducted herself properly, and has kept up the payment of her own premium. We reckon, therefore, that with a salary of £45, uniform to the value of £4, and £7 towards a pension and a bonus, our private nurses are liberally treated."

"You came here from London, I think?"

"From the Royal Hospital for Children and Women, where I was matron from 1896 to 1899. I shall have been at Reading three years next month. I was trained at the Nightingale School, and afterwards I was ward sister at St. Thomas's Hospital.

### The Nurses at the Paddington Green Children's Hospital.

By a Correspondent.

Under the heading "Overworked Nurses: the Weary Round of Hospital Life," one of the London daily papers recently published particulars of the hours of work at the Paddington Green Children's Hospital, adding severe strictures on what was described as a "system of slavery." The following particulars, the matron told a correspondent, are correct:—

On day duty the nurse's work begins at 7 A.M. At that hour she has breakfasted, and is in the ward. She remains on duty for eleven hours, except two intervals for meals of half an hour each. This is a daily record for a working week of seven days. Two hours off duty are allowed after work. These may be given in the middle of the day, in which case the work beginning at 7 A.M. is not over till P.M. Night duty starts at 8 P.M. and lasts till 7 A.M.

The manner in which the statements made by the matron to the representative of the paper in question were distorted, has been a source of some annoyance both to the matron herself and to the hospital committee. It has been decided not to supply any information to inquirers except through the committee, and not to correct the errors made by the paper referred to, but to let the matter drop. Three points, however, came out in the course of conversation with our correspondent: (1) The nurse who was stated to have said she had not been out for ten days added that it was her own fault, and while the first fact was made the most of, the latter was suppressed entirely. A rule has since been made with regard to going out, which was before an understood thing, but not obligatory. (2) The probationers' hours have not, as was stated, been lessened by one and a half hours a day since the new matron's arrival. And (3) the matron herself does not "start at 8 A.M. and finish at 11.30 P.M." without an interval. Miss Pinchard's private opinions as to nurses' hours generally do not, she said, affect the question; and she is quite ready to admit that, with more liberal public subscriptions, it might be found advisable to add to the nursing staff. "If," she said, "I had another bedroom, I might take another probationer." The matron added that the health of the nurses at Paddington Green is exceedingly good, and that she has heard no complaints from them about the long hours.

### Sick-Room Cookery.

By Maude Mason, Principal of the Bradford School of Cookery.

### PEPTONISED AND MALTED FOODS.

(*Continued from page* 216.)

It is sometimes necessary, owing to the feeble condition of the digestive organs of a patient, to administer foods which have been previously partly, or entirely, digested. The length of time these preparations should be given will be specified by the medical man; if too long used the digestive organs become enervated, and do not regain their activity. There are three methods employed of partially digesting food before it is taken into the stomach:

1. The addition of "malt extract" or malt meal.
2. The addition of pepsin (derived from the gastric gland of certain animals).
3. The addition of pancreas extract.

We will consider them in their turn.

### Malt Extract.

During the process of digestion starchy foods are acted upon by a ferment in the saliva and pancreatic juice which converts the starch into dextrin and sugar (maltose); it is absolutely necessary that this change in the starch should take place before it can be assimilated. Malt, which is prepared from barley, contains a substance, diastase, which has the same power as the ptyalin of the saliva and the amylopsin of the pancreatic juice; that is, when mixed with starchy foods it converts them into dextrin and sugar, and thus it has received the name of "animal diastase." Therefore, it will readily be seen that the use of malt is restricted to association with starchy foods, that is, particularly cereals—as wheat, oatmeal, cornflour, rice, etc. The use of some of the bought malted foods by themselves, merely mixed with water, is not recommended, but if mixed with gruel, etc., are valuable, provided they still contain active diastase. Malt extract or malt flour may be used to bring about this result. The extract may be bought ready made, or may be prepared by soaking crushed malt in cold water; let it stand over night, then strain (Sir William Roberts' method). To malt gruel, make a gruel in the ordinary way, but thicker, strain it, and to every pint, add two tablespoonfuls of the extract. Notice that the gruel becomes thinner and sweeter. Rice pudding, oatmeal porridge, pea soup, etc., may be treated in a like manner. Malt flour may be used in a similar way, and by many authorities it is considered superior to the extract. A teaspoonful is sufficient to add to a rice pudding made with 1 pint of milk and 2 ozs. of rice. Bear in mind the following points: the malt extract or flour should not be added until the food has been cooked; the temperature of the food should be allowed to fall until it is about the right temperature to swallow before the malt is added (all ferments act best at a temperature under 150° F.); the malt will thin and sweeten the food, extract and flour should both be fresh when used. Some of the malted foods on the market which contain ground malt, wheat flour, potassium, bicarbonate and milk are highly nutritious. Directions in most instances accompany them.

### Pepsin Preparations.

These are most often used, not for pre-digesting foods, but as an addition to the food to stimulate the gastric digestion in the stomach. Several satisfactory preparations are to be obtained.

### Peptonised Foods.

The object in peptonising food is to relieve the work of the stomach by converting insoluble albumen into diffusible peptones before they are taken into the stomach. This might be done with pepsin and hydrochloric acid, but it is much more usual to employ a pancreatic extract, etc., because the latter is equivalent to the work of the pancreatic juice, which is the most powerful digestive agent in the body. Its action is, however, destroyed in an acid medium, as by the hydrochloric acid of the gastric juice; it is, therefore, obvious that the food must be pre-digested by the pancreatic extract before it enters the mouth. Peptonised foods are very suitable for rectal alimentation. The action of the pancreatic juice is fourfold: it not only converts albumen into peptones, it also converts starch into sugar, splits up fats and curdles milk, therefore it is more valuable than merely pepsin, which only, acts on albuminates. Several varieties of foods may be bought already peptonised, but they may easily be prepared by means of pancreatic extract. It is desirable, however, when food is completely peptonised to use some flavouring to conceal the otherwise unpleasant taste. To peptonised milk a little coffee added is an improvement, and beef teas and broths should have some vegetables boiled in them; a pinch of celery seed or a tiny piece of fried onion is wonderful for disguising the flavour.

### Using the Extracts.

Directions for the use of the extracts are usually supplied with them. I might, however, mention that peptonised gruel may be used instead of water for making soup from beef, chicken, etc. Peptonised milk may be converted into blanc mange by the addition of ¼ oz. isinglass (previously soaked) to ½ pint. peptonised milk, which must be boiled before the isinglass is added, or it will not set; stir over the fire till the isinglass is melted, add a little cream and flavouring, and put into a cold place to set. Peptonised gruel may be converted into a jelly in a similar way. The addition of grated lemon rind, cinnamon, or little brandy or sherry is an improvement.

### The Royal British Nurses' Association.

Lecture by Sir T. Lauder Brunton.

On Tuesday afternoon Sir Thomas Lauder Brunton lectured at 10 Orchard Street on "Spiritualism and Hallucinations." There was a very full attendance, and late comers were obliged to be content with standing-room outside the door.

The lecturer dealt with "ghosts" and visions, many of which, he said, were the result of an epileptic condition, while others might be caused by hallucinations arising from disease of the eye, of the nerve connecting it with the brain, or of parts of the brain itself.

He had been much criticised for speaking, in a previous lecture, of the feats of Samson as those of a man subject to epileptic fits; in his opinion there was much that pointed to that conclusion, and the "shaking" by which Samson was wont to recover his strength may have been not unlike the contortions of the howling dervish who occasionally falls down in an epileptic fit. There could be no truer description of such fits than the account of Mahomet "foaming at the mouth and bellowing like a bull." What was wanted was to reduce as many of such phenomena as possible to scientific basis, and this was his object in giving the lecture

### A Fragment.

Poor little pro.!
In uniform so evidently new,
With grimy hands, and snowy cap askew,
With tear-stained face and many a mournful sigh
You bend o'er tins that stubbornly defy
Attempts to make them shine.

And I, the grey-haired sister, little pro.,
Noting your doleful figure as I pass,
Awhile muse on the magnifying-glass
Through which, in youth, we view each tiny woe.

*Elyadin Luce*

### The Hospital for Animals, Bombay.

A VISIT BY A SISTER.

LOVERS of animals may be interested in a brief description of "The Bai Saharbai Petit Hospital," in which our four-footed friends are treated and nursed with more of scientific skill and tender care than was available for the natives of this city little more than half a century ago. The hospital was built in the year 1893, at very considerable cost, and subject to certain conditions was presented to the Bombay Society for the Prevention of Cruelty to Animals, one of these conditions being that the institution should be called after the wife of the donor. The Government Veterinary College is located in the same compound. By a special arrangement with Government the services of the teaching staff are at the disposal of the hospital, which in its turn affords every clinical facility to the large number of students attending the college.

THE WARDS.

The entrance to the hospital grounds is through a lofty arched gateway erected by Mrs. Dinabai Petit in memory of her son. On the top of the arch, on one side, is a life-size figure of a bullock, flanked on the other by that of a horse, whilst between them, in an attitude that can only be described as "perky," its ears erect and tail well curled, stands a little dog. In the arched recess immediately underneath the dog, in bas-relief, is represented a country cart drawn by bullocks, with its attendant cart-man. The hospital is most complete in every way. The long and lofty wards are beautifully ventilated. The stone floors slope downwards from the walls to a narrow drain which runs right through the centre of each building. This drain is constantly flushed out and the wards are thus kept perfectly sweet and clean. Besides the four wards for cattle, two for horses, and one for dogs which form the main part of the hospital, the buildings comprise an isolation ward, tetanus ward, dark room, post-mortem room, office, dispensary, and forge. Operations are performed in special enclosures, one being for bullocks, the other for horses. In the dog ward two cells are set apart for dogs suffering from rabies. The rules provide for the prompt admission of patients "at all hours of the day and night," and it is added that "emergency cases may be brought at any time." There is an out-patients' department open daily (Sundays excepted) from 10 A.M. till 12 noon. The nursing of the sick animals and the dressing of their wounds is entrusted to the students, two of whom are always on duty during the day and one during the night. They are assisted by the ward-attendants, namely, syces and dog-boys. In company with a friend I visited the hospital one Saturday half-holiday, when no classes were being held in the College. A Goanese student, in the third and last year of his training, showed us over the buildings, and was at considerable pains to explain the various arrangements to us.

THE DIET SHEET.

First we were taken into the store-room to inspect the various grains on which the animals are fed. The store-keeper is a Parsee, and his pretty children came to peep at us. They made a picturesque group in their bright garments, which enhanced the beauty of their olive complexions and large, dark eyes. From the store-room we went to the cattle wards. It was feeding time, and the poor patient old biles (bullocks) were enjoying their evening meal. The greater number of them were suffering from sores and wounds. We were amused to see the diet sheet of each patient duly framed, and hanging in his stall.

THE USE OF CHARTS.

The horses were next visited. We found them much more interesting than the bullocks. Each is provided with a commodious loose-box, but nearly all wore heel ropes lest they should rub, and so reopen wounds that were healing, or pull off their bandages. One horse, suffering from broken ribs, was slung up by means of a broad bandage passed under his chest, another wore a large necklace or collar made of pieces of bamboo, which effectually prevented him from licking the wound on his flanks that was being treated with some poisonous drug. As he could not bend his head, his supper was comfortably arranged for him in a little hammock just under his nose, and he seemed to be having a pretty good time in spite of the troublesome collar. In the horse and dog wards the fever cases have charts for the registration of temperature, pulse, and respiration. We were shown the chart of a horse that had had several days' fever. We noticed that the highest temperature marked was 103·4. The student who accompanied us showed us how to take the horse's pulse, by pressing lightly one of the arteries in the throat. The pulse of a dog, he told us, is felt in the paw just below the claw. The canine patients were few in number, each doggie had a separate cell or room enclosed in iron bars and furnished with comfortable little beds on platforms. Of course they welcomed us warmly, poking their glossy muzzles through the bars, striving to lick our hands with their red tongues and wagging their tails at a great rate, trying to tell us in their own dog language how weary they were of their incarceration. But there was one, a brindled hound, who had no greeting to give and who was quite unconscious of our presence as he lay, breathing painfully, on his straw pallet. "That dog is very bad," remarked the student, and indeed the poor beast seemed almost *in extremis*. The isolation ward for contagious diseases had a great many inmates, skin eruptions being very common among bullocks. This ward is most carefully and constantly disinfected.

CASES OF SUNSTROKE.

Horses in Bombay suffer much from sunstroke. As a means of prevention, careful owners provide their steeds with sun hats to be worn during the heat of the day. These are often covered with white calico covers, trimmed with a jaunty little frill at the back, but this frill is no mere ornament. It serves to protect the neck of its wearer from the tormenting horse-flies. We were told that horses brought to the hospital suffering from sunstroke usually recovered quickly with the rest and quiet provided by the dark room. A peep into the dispensary brought this interesting visit to a close. We gleaned here an interesting piece of information regarding the relative doses of medicine administered to different animals. A dog is given a dose as nearly as possible approximating to that given to a human being, whilst a horse requires sixteen, and a bullock twenty times as much.

### To Nurses.

**WE invite contributions from any of our readers, and shall be glad to pay for "Notes on News from the Nursing World," or for articles describing nursing experiences, or dealing with any nursing question from an original point of view. The minimum payment for contributions is 5s., but we welcome interesting contributions of a column, or a page, in length. It may be added that notices of appointments, entertainments, presentations, and deaths are not paid for, but that we are always glad to receive them. All rejected manuscripts are returned in due course, and all payments for manuscripts used are made as early as possible after the beginning of each quarter.**

### Lectures to Head Sisters.

By E. MARGARET FOX, Matron of Tottenham Hospital, N.

LECTURE III.—(*Continued from page* 217).—A HEAD SISTER'S DUTIES TOWARDS HER PATIENTS.

IN certain cases, as of hysteria and other mental diseases, the value of moral treatment is openly recognised by doctors. That neurotic girl whose thoughts are entirely centred on herself, who simulates disease and refuses food in order to obtain sympathy. How are you to treat her? It is here that your moral influence will greatly tell. A quiet, kind, but not too sympathetic manner—a firm insistence as a matter of course on food being taken, and any ordered treatment being carried out—a judicious leading of her thoughts out of and away from herself and her own interests, all help to restore the ill-balanced mind to its normal state of health.

Books, too, will aid you very much in giving a favourable direction to your patients' thoughts. Nowadays, when everyone reads something, books and papers help most pleasantly to pass the long hours of convalescence, and by taking a little trouble, you can do much towards providing good and suitable reading for all your patients. Let the little ones in the children's wards have their picture books at stated times, and hold them out as rewards for being good during trying periods of painful dressings. They will value them far more if you set a certain value on them as well, and do not allow them to be wantonly soiled and torn to pieces. The toy and book cupboard can be made to exercise a wonderfully salutary influence both on mind and morals if the children are made to understand they can only have the things as rewards for good behaviour. It is well to remember in dealing with children that encouragement to do right is far and away better than prohibition of what is wrong, and that a constant succession of "Dont's" make a child's life miserable, while our aim should be to make them happy. The children's relatives often seem to think that if only a book has pictures in it every requirement of the child's mind will be satisfied; like the father of one little boy of five, who each visiting day used to bring him in the *Illustrated Police News*, and unfold with pride and admiration its dreadful pages depicting murders, suicides, wife-beatings, and all kinds of horrors! Imagine the mental impressions produced by such pictures on a child's vivid imagination and large imitative faculties.

And because most boys like tales of travel and adventure, and stories with a comic element, that is no reason why they should confine their reading entirely to journals of the halfpenny press. See that they get good magazines and encourage them to read some of the many excellent boys' books that most hospitals have given them from time to time.

In the men's ward, while nothing will ever take the place of the daily paper, when that is read, study their individual tastes and try to develop them in the right direction. The girls and women generally read a great deal, and evince much fondness for halfpenny novelettes, dress and fashion papers, and semi-religious stories. Sometimes you will find that good children's books are much liked by grown-up people, and can be taken in easily at a time when the brain, weak from recent illness, would be unable to grasp anything deeper. Try to suit their reading to their various tastes, capacities, and temperaments. Give something bright and cheerful, funny even, to that patient suffering from nervous depression, or awaiting an operation; large typed books, simply worded, to the aged, and more solid reading to the better educated. Encourage the talkative ones to read instead, especially during the hour or two after dinner, when some like to have a nap, and do not want to be disturbed by the needless chatter of one or two convalescents.

Not only the books, but your own attitude towards your patients must be subject to variation according to their requirements. Different types of people need different ways of being dealt with. Take, for instance, the grumbling, exacting patient, which is a troublesome and well-known type. You will find it best to place such either with an empty bed on each side of him, or next to the most cheerful patient in the ward. Never put two grumblers side by side, if you ever are so unfortunate as to have two at once of this variety. Always be careful to give them no just cause of complaint against you, but do not favour them in the least. Have little to say to them, and be uniformly kind and obliging. Sometimes these cases are those who imagine they have not been well treated in some other hospital where they were before, and will soon become quite pleasant after they once get used to your methods. Never allow yourself or your probationers to quarrel or contend with a patient.

Be quite sure you are in the right in what you require of him, and if he positively refuses to do as you ask him quietly give up the struggle, and report the fact. The excitement and anger produced in his mind by his opposition will do him far more harm than the disputed dose of medicine or feeder of milk will do him good. But, unless they are delirious, you will not often find this happen among adults, for, as a rule, they feel themselves neglected if medicine is not prescribed for them, and the more nauseous the dose the more "strengthening" they suppose it to be.

To aged patients, let your manner be marked by rather more consideration than to younger persons. Give them their own way if possible, and do not argue with them. Also, beware of offending them by calling them all "Grannie" or "Daddie" indiscriminately, some old people being very sensitive on the subject of their advancing years, and not liking to be reminded of it. Especially, do not be incessantly talking to any of your patients. It is apt to worry them, and they ought never to have to wish that sister would be quiet and let them have a little peace.

Shy, nervous and despondent patients need a little extra cheer and encouragement. Do your best to make such feel at home, and anticipate their wants so as to save them the pain of asking strangers for small services.

In a medical ward the patients on admission generally feel, as they express it, more "bodily ill" than surgical cases. They should be quickly and quietly put to bed as soon as possible, and not talked to more than is absolutely necessary. An effusive welcome, a noisy greeting, would be quite out of place here. Put them at once into the best position for absolute rest, and then apply the other principles of good nursing to them, providing them with good air, suitable food, a clean skin, and warmth.

### Death in Our Ranks.

WE record with regret the death of Nurse Patterson Hudson, which took place on the 15th inst. from enteric fever contracted by her in the discharge of her duties. Miss Hudson joined the Nurses' Home, Granville Road, Newcastle, in 1898, and was trained at the Brownlow Hill Infirmary, Liverpool, subsequently working as a private nurse. The funeral was attended by many friends, and several of her fellow nurses were present with the matron and assistant of the home. Many beautiful wreaths were laid upon the grave.

### "Nurses in Kilts."

A RIDICULOUS STORY.

AT the meeting of the Greenwich Board of Guardians last Friday, the chairman of the infirmary committee was asked "if he was aware that a fancy dress ball had been held at the infirmary recently, at which some of the nurses appeared in male costume, one of them being in kilts." The chairman said he was not aware that any such festivity had taken place, and asked that the question might be repeated. Meanwhile, one of our representatives called to see the matron, who stated that no fancy dress ball had taken place in the infirmary. "The fact is," she said, "that some of the nurses in their recreation time one evening, dressed up in various costumes. I am not aware that any special characters were chosen. They had done it before, implicitly obeying my directions that great care should be taken not to appear in any of the corridors, and to wear cloaks if obliged to be seen outside the recreation room. They are very young, some of them mere girls, but I have a particularly nice set of nurses, and I know they were careful; they assured me that on the occasion referred to, all the blinds were drawn down. The recreation room is in an unfortunate position, but still I cannot think that they were overlooked from outside, and I am entirely at a loss to account for the matter having become public. As to the 'nurse in kilts,' one of the nurses, with a patient's shawl round her, much too long to be smart, represented a Scotch lassie, not a man at all. On behalf of the nurses and the training school, myself and the profession generally, I intend to protest against this scandal at the Board's meeting on the 30th."

It seemed very hard, Miss Dixon said, after four years' strenuous work to bring the training school up to modern requirements, that the nurses' names should be dragged through the mire in this way, and personally she felt it more keenly than she could express, and her feeling was shared by the Infirmary Committee.

"As a matter of detail," she added, "a fancy dress ball could hardly have been held in the Infirmary without the knowledge of the chairman of that committee, and THE HOSPITAL will be doing us a great service by authoritatively contradicting the statement that any such festivities took place here."

### Everybody's Opinion.

[Correspondence on all subjects is invited, but we cannot in any way be responsible for the opinions expressed by our correspondents. No communication can be entertained if the name and address of the correspondent are not given as a guarantee of good faith, but not necessarily for publication. All correspondents should write on one side of the paper only.]

### FRICTION AT A CAPETOWN HOSPITAL.

"A NURSE" writing from New Somerset Hospital, Capetown, December 24th, 1901, says:—In your note of November 23d, you refer to the friction which exists among the nursing staff of the New Somerset Hospital, Capetown, I wish to state that the friction is not among the nurses at all, and is not caused by the nursing accommodation. I have been in several hospitals during my nursing career and have never found such good fellowship amongst the nurses as I have in the New Somerset Hospital. The friction is caused by the general upsetting of all the old rules and regulations. In consequence of these changes the matron has been forced to resign, which action has been greatly deplored by a large majority of the public of Capetown, by whom she is universally respected. In every hospital there is room for improvement, but there are other ways of attaining that object than those chosen by the medical officer. I would be glad if you would insert this in your paper.

### NURSES' HOLIDAYS.

"EGLANTINE" writes: A nurse who has been working hard all through the long year at last gets off for a holiday. When she returns all the nurses tell her how much better she looks, and she feels better; but any thoughtful person will confess that two weeks and two days is not sufficient holiday for a hard-worked nurse. I know a doctor who says that if only he had time to spare he would write to the papers on the subject of the shortness of nurses' holidays. Surely it is time that something was done. In one London hospital the probationers only get two weeks' holiday and two days for travelling, the staff nurses get three weeks; but would a month be too long for them? At other hospitals the probationers get three weeks. When we think of how they work for 10 and 12 hours a day we must realise that they need a good change and rest. No doubt most hospitals are terribly undermanned and therefore it is difficult to allow longer holidays; but cannot something be done to remedy this great evil?

### "OVERWORKED NURSES."

"NURSE ALICE" writes: On duty at seven o'clock, I rush' probably half famished, to join my comrades at bed-making The cold is soon forgotten talking about it, and by the time dusting is over I am too warm. Everything must be quite ready by the time we hear the step of our universal favourite —the house surgeon. We all walk after him while he makes his morning inspection of our wards. Luncheon then is served to patients, and we have some, too, if we had not time before nine o'clock to get any. Lockers are scrubbed, brasses and tins cleaned, patients' wants fill up the time until dinner comes from the kitchen, and then we all take the dinners round—a laugh here, a cheery word there, "Why Mrs. D. you are promoted to fish to-day!" and so on. Afternoon finds you perhaps still "on duty," through someone knocking someone else down, and so filling your accident-bed. Still, there are ups and downs in all working women's lives, and there is no need to grumble. You get off duty at perhaps eight o'clock, but it may be nine o'clock if any patient is very ill, and then comes supper, a gossip, and to bed. Bed, I have heard a nurse say, was her idea of a holiday. These people who measure up time and the amount of work done quite forget also to measure the amount of happy and congenial companionship gained, or to remember that these nurses chose the work themselves. Absence of worry should, too, count for much, for, unless they worry themselves—some people always can make "a worry"—there should be none, and mental strain should be unknown to the nurse with an easy conscience, who does her duty to patient, doctor and matron.

### OPEN-AIR TREATMENT AT STAPLETON INFIRMARY.

"NURSE J." writes: Thanks to the energy on the part of the medical officer, a long-felt want has at last been realised in Bristol, and those poor consumptives who are unable to obtain admission to an open-air sanatorium are enjoying all the privileges of the open-air treatment at Stapleton Infirmary. In addition to a liberal meat diet, each patient receives two quarts of milk and an egg per day. Even the comfort of the pipe is not denied them, each man being on a weekly allowance of tobacco. There are at present 30 patients under treatment, and there is already a marked improvement in those in whom the disease is not too far advanced. Those patients who are strong enough enjoy two country walks a day, one from 10 to 11.30, the other from 2 till 3.30. I am sure we must all hope—at least those of us who take any interest in our poorer brethren—that before very long many of these patients may be able to resume their ordinary occupations, and that with all the improvements in medical science England's most fatal disease may be stamped out of our midst.

### Appointments.

City of Glasgow Fever Hospital, Ruchill.—Miss Edith M. Harrison has been appointed staff nurse. She was trained at Plaistow Hospital.

Hastings Borough Sanatorium.—Mrs. Annie Ellis has been appointed matron, and Miss Mabel Cribbs and Miss Jessie Joel have been appointed charge nurses. Mrs. Ellis was trained at the Royal Infirmary, Derby, and has since been night superintendent at Fountain Fever Hospital, Tooting; matron of the Infectious Diseases Hospital, Stratford-on-Avon; and matron of the Sisters' Hospital for Infectious Diseases, St. Albans. Miss Cribbs was trained at Belvedere Hospital, Glasgow, for three years, where she was afterwards charge nurse for twelve months. She has since been private nurse at the Sunderland Nursing Institute for fifteen months, and private nurse on her own account in Glasgow. Miss Joel was trained at Hove Borough Sanatorium, and has since been engaged in private nursing.

Memorial Hospital, Buluwayo, Rhodesia. — Miss Ethel Macfie has been appointed matron. She was trained at St. Marylebone Infirmary, London, where she afterwards became sister. She has since been charge nurse at the Eastern Fever Hospital, Homerton; night superintendent, and afterwards matron at a Capetown Plague Hospital.

Monkwearmouth and Southwick Hospital. — Miss Edith Rose Jarrett has been appointed staff nurse. She was trained at the New Hospital for Women, Euston Road, London, and the National Orthopædic Hospital, Great Portland Street, London, and has since been nurse at Gravesend Infirmary.

Norfolk and Norwich Eye Infirmary.—Miss Madeline Whitehouse has been appointed matron. She was trained at Westminster Hospital, where she was afterwards charge nurse of the surgical wards for a year. She has since been matron at the Cheltenham Eye, Ear and Throat Hospital.

Rotherham Hospital.—Miss Maude A. Buckingham has been appointed matron. She was trained at St. Bartholomew's Hospital, London.

Royal Infirmary, Hull.—Miss Maud Crichton has been appointed night superintendent. She was trained at the Leeds General Infirmary, in which institution she was sister for two years, and did holiday duty as assistant matron.

Royal Portsmouth Hospital.—Miss May Reynolds has been appointed night sister. She was trained at the Royal Albert Edward Infirmary, Wigan, and has since been nurse at the City Hospital, Liverpool; night sister at the Grimsby and District Hospital; and temporary night sister at the Royal South Hants Hospital, Southampton. Miss Reynolds, who holds the L.O.S. certificate, has also done private nursing.

Royal Victoria Hospital, Bournemouth.—Miss Edith M. Wheeler has been appointed charge night nurse. She was trained at the Royal Victoria Hospital, Montreal, Canada, and has since done private nursing and district work. She has also been temporary night superintendent at the Royal Isle of Wight Hospital, Ryde.

St. Mary Islington Infirmary, Highgate Hill, Holloway.—Miss Louise Thurling and Miss Beatrice Price have been appointed sisters. Miss Thurling was trained at Southwark Infirmary, East Dulwich, and has since been charge nurse at the Grove Fever Hospital, Tooting, and private nurse at Mentone. Miss Price was trained at Portsmouth Infirmary, and has since been staff nurse at Highgate Hill Infirmary.

Southwark Infirmary, East Dulwich Grove.—Miss Frances C. Wallen has been appointed night superintendent. She was trained at the Metropolitan Hospital, London, holds L.O.S., and for the last year has been sister at Southwark Infirmary.

Stockport Infirmary.—Miss Adelina Stuttard Aynsworth has been appointed night sister. She was trained at the Infirmary and Dispensary, Bolton, afterwards holding a post in Dr. Tapp Sinclair's private hospital at Manchester. Subsequently she was night superintendent, sister of male medical, surgical, and ophthalmic wards, and sister of operating theatre, out-patients' department, accident room, and women's wards at Bolton Infirmary; as senior sister she took matron's holiday duty. She has since been sister of women's medical, surgical, and gynæcological wards at the General Infirmary, Macclesfield.

Walsall Victoria Nursing Institution. — Miss Elizabeth Holloway has been appointed lady superintendent.

### Presentations.

Cardiff Infirmary.—Sister Wadham, who was trained at Cardiff Infirmary, and has been on the nursing staff ever since that time, has been presented by the chairman, on behalf of himself and several members of the Board of Management, with a handsome travelling bag, in connection with her departure from Cardiff to serve in South Africa.

St. John's House Nursing Association, Worcester.—Miss M. E. Caldecot, who is leaving Worcester to take up an appointment at the Voluntary Hospital, Barry Dock, has been presented with a very handsome wallet completely fitted up, and bearing a suitable inscription, in token of the love and esteem in which she was held during the two years of her district work in connection with St. John's House Nursing Association, Worcester.

### Where to Go.

The New Gallery.—The exhibition now on view at the New Gallery is one of unusual interest. The catalogue is so full of delightful historical notes concerning many of the exhibits that even apart from them it is excellent reading. The arrangement of the galleries helps to take us in chronological order from the early Kings of England to the present day. As the catalogue explains, the object of the collectors has been to help the public to follow the development of the British Monarchy. The complete collection of Royal portraits assists us to understand the personal influence exercised upon his generation by each individual sovereign. The objects appertaining to Royalty and State of the different periods lend colour and reality to the times they illustrate. Here is a wealth of beautiful workmanship and interesting curios in the glass cases which fill the galleries, far more than we can even mention in the briefest manner, and it is most difficult to select any one thing for comment when so many seem equally to claim attention. The Book of Hours of Mary Queen of Scots, said to have been used by her on the scaffold; one of Henry VIII.'s characteristic hats; an autograph letter from Queen Katherine Parr to Lord Seymour accepting his offer of marriage; a purse worked by Queen Elizabeth; a portion of one of Prince Charles Edward's tartans; a letter of Charles I., and of many things of more modern interest, should not be missed. The collection of miniatures and autograph letters is unusually fine, and the whole exhibition is one worthy of a leisurely inspection, which it will amply repay.

A ball in aid of the Church Lads' Brigade drill and night club rooms in the East End will be given on February 7th at the Grafton Galleries. Tickets (12s. 6d.) to be obtained from the honorary secretary, Mrs. Harvey, 92 Lexham Gardens, S.W.

### Wants and Workers.

Nurse Stockwell returns her thanks to the kind friends who so promptly responded to the appeal made last summer for old rags for a poor man with cancer. No more will be needed the patient has recently died.

### A Book and its Story.

### COUNTRY SOCIAL LIFE IN THE LAST CENTURY.*

THE authoress of "Links with the Past," whose chain of memory extends from the early days of the last century to those of its close, has had from her social position exceptional opportunities for storing up memories, which go to make the unusually interesting volume reminiscences before us. A valuable addition to these glimpses at the past is made by the inclusion of extracts from the journals of Miss Mary Bagot, which carry the reader back into the eighteenth century and give a vivid impression of country social life, and of society at large in those days. Mrs. Bagot was born in 1821, and married Captain, afterwards Colonel Bagot, of the Grenadier Guards, in 1846. Her father, Admiral Percy, was a distinguished naval officer, whose twin brother became in 1831 Bishop of Carlisle. His elder brother succeeded to the Dukedom of Northumberland on the death of the fourth Duke. The childhood and early years of the authoress were wandering ones. She accompanied her father on his voyages on more than one occasion; and being quick of observation, and not without a sense of humour, nothing was lost to her perceptive vision. One of her first recollections is that of the voyage to Ireland in 1829. Captain Percy had been given the command of the Royal yacht *Charlotte* at Kingstown. Upon the death of George IV., in the following year, they returned to England, where they arrived in time to see the first train started from Watford to London. "The excitement caused seems inconceivable in these days. . . . People went to discuss and compare their sensations after their first railway journey, and would solemnly ask each other whether their hearts and breathing were not affected by the rapid motion through the air. . . . Old gentlemen predicted the downfall of the country, at dessert, over their port wine, and I remember listening awestruck, and secretly longing intensely to travel by train, which I very soon did. A report spread abroad that Lord Grey and Lord Brougham were going to be taken to the Tower. My brother and I walked miles from Scotsbridge to Watford to see them leave, from whence we believed they would start for the Tower, where we devoutly hoped they would be beheaded."

In 1842 the authoress accompanied her father, when he took the command of the Cape of Good Hope Station, on board his flagship, H.M.S. *Winchester*. During their sojourn they made many excursions up country. In those days there were no hotels, and travellers were indebted for their accommodation to Boer farmers, who, upon payment, would take them in for the night. Sixty years ago the Boer farmer and his vrow differed little from their descendants of to-day. Even the very names of places were typical—for it was in the vicinity of "Wait-a-bit-Valley" that the travellers sought shelter at one of the farms. The owner was very grumpy and rude, and bedrooms would willingly have been exchanged for the bare ground. The beds were horribly stuffy, and the rooms smelt of cockroaches. "The fat house Frau had been really very kind, but she was enormously fat and lost in astonishment at the slim English girls, of whom she made many inquiries; the one in which she was most interested being, 'Why are you so thin?'" Other experiences were more pleasant; for instance, "Zandoliet, a delightful farm and house belonging to the Cloetes, was most comfortable, and the family who owned it most charming. It was very interesting to see all the vast herds of cattle and ostriches go out in the mornings and return in the evening to the farm. We used to dance every evening, and before dinner Mr. Lawrence Cloete used to stand on his doorstep, put his hands to his mouth and give a tremendous 'view holloa' in case any traveller had lost his way—true patriarchial hospitality." One other specimen of a Boer host is given. Riding parties frequently went out to breakfast from Capetown to Kalks Bay, seven miles distant. Here they were received by "Farmer Peck, an old rogue, if ever there was one, but very amusing. I believe he had been a famous smuggler. I remember asking him for whom he was building a house across the road, immediately opposite his own. He replied, with a wink, 'For Mrs. Peck when she requires change of air.'" Among other experiences is the pursuit of a slaver on the West Coast of Africa. She was captured, and the ladies from the *Winchester* boarded her. No stronger contrast could be offered than that between the Spanish captain's quarters, in which "a guitar with blue ribbons was found, nice books and every luxury," and those of the poor slaves. "The slave deck was an awful sight. How human beings could be packed into it was marvellous and horrible. They were doubled up, their knees meeting their chins. Twice a day the poor wretches were ordered up on deck that they might not die, as many tried to do; and if they would not walk and stand upright they were flogged until they did." The slaver was condemned and was sent to Sierra Leone, where the slaves would be set free and made apprentices. "If apprenticed to Boers they were more cruelly treated and regretted the days of slavery and good masters." To return to our own country, there is a charming description of Blithfield, the family place of the Bagots, and it is delightful to read in these days of change, "that Bagot's Park and the surrounding estate were in the family previous to the coming of William the Conqueror, and the family holds them still." The great features of Bagot's Park are the oaks. One, "The Beggar's Oak," mentioned in "Doomsday Book," is still a mighty tree: the girth of its trunk so large that a carriage and four horses can be almost concealed from view when drawn up behind it. The most curious extract from Miss Mary Bagot's journal is a genuine ghost story which must conclude our notice of these chatty reminiscences. Had it been told first-hand, perhaps, we should have been more inclined to believe it, and yet—who can doubt the testimony of "a maiden lady of perfect credulity," and one of the two concerned in it, from whom the narrator, a clergyman, received it? The sisters lived alone in their home, a large house in Ireland. One winter's night when seated near the fireplace in the library the younger sister, having left the room for a moment, the elder one heard steps; the door opened, and her father entered. He had been dead some years. "In an awful and indescribable manner I *felt* that he passed by me to his accustomed chair by the fire. Again I heard footsteps, I could neither move nor speak. This time it was my sister who entered. She screamed, and the candle fell from her hand. I rushed forward, 'What do you see?' I asked. She replied, 'My father is in his chair.' The next moment he had vanished from our eyes." The anecdotes quoted do not in any way represent adequately the more important matter of which the book is full, viz.—that having reference to contemporary, historical, and political personages, and events. For these we must refer the reader to the book itself. A charming portrait of Mrs. Bagot, from a painting by Manteroni, shows a face of much beauty and refinement; and a quaint picture of Miss Mary Bagot adds to the interest of the volume.

* "Links with the Past." By Mrs. Charles Bagot. (Publisher, E. Arnold: London. 1 Vol. 16s.)

### For Reading to the Sick.

"THE FRUIT OF THE SPIRIT IS . . . JOY."

In every gladness, Lord, Thou art
The deeper joy behind.—*Macdonald.*

The heart that trusts for ever sings,
And feels as light as it had wings;
A well of peace within it springs,—
Come good or ill,
Whate'er to-day, to-morrow brings,
It is His will.—*Rev. Isaac Williams.*

I thank Thee, too, that Thou hast made
Joy to abound;
So many gentle thoughts and deeds
Circling us round,
That in the darkest spot of earth
Some love is found.

For Thou Who knowest, Lord, how soon
Our weak heart clings,
Hast given us joys tender and true,
But all with wings,—
So that we see, gleaming on high,
Diviner things.—*A. Procter.*

True joy is not like animal spirits, which rise and fall, but it is a gift which we must possess always, not merely in prosperity but in adversity. It is only when a soul has completely accepted the truth that every event, great or small, which occurs in our daily life has been planned by a loving omniscient Father, who is ever working for His creatures' good, that it is able to "rejoice in the Lord alway."

*T. B. Dover.*

It makes all the difference to life if Joy is shining within. It sheds a rainbow light across the darkest storm. And brightness not only makes a difference to our own lives, but also to the lives of other people, if instead of the creaking, groaning machinery, they have in its place the smooth, easy, joyous life before their eyes. Bright lives do a great deal to cheer and help all around them. Perhaps others are bearing their cross better, or doing their work with greater ease, because they can walk in our brightness; whereas gloom and melancholy, and "the indolent rebellion of complaint," would cause them to loosen their hold from very weariness, and to fall crushed and broken. In every life which is working harmoniously there will be the strong impress of Joy, as the fruit of the Spirit growing up in the soul which is alive unto God.—*Canon Newbolt.*

Poor fainting spirit, still hold on thy way,
The dawn is near!
True, thou art weary; but yon brightening ray
Becomes more clear.
Bear up a little longer—wait for rest—
Yield not to slumber, though with toil opprest.

"Joyful through hope" thy motto still must be;
The dawn is near!
What glories will that dawn unfold to thee!
Be of good cheer!
Gird up thy loins, bind sandals on thy feet;
The way is dark and long, the end is sweet.

*Charlotte Elliott.*

### Notes and Queries.

**The Editor is always willing to answer in this column, without any fee, all reasonable questions, as soon as possible.**
**But the following rules must be carefully observed:—**
**1. Every communication must be accompanied by the name and address of the writer.**
**2. The question must always bear upon nursing, directly or indirectly.**
**If an answer is required by letter a fee of half-a-crown must be enclosed with the note containing the inquiry.**

*Superannuation.*

(155) Having served 13 years as nurse in a workhouse infirmary and six years in a fever hospital, both under the same Board of Guardians, am I entitled to the benefits of the Superannuation Fund or to a pension of any kind?—*Pension.*

You had better write to the Local Government Board, Whitehall, and state your case, which doubtless comes under the provisions of the Poor Law Officers' Superannuation Acts, 1896 and 1897. Of course, we conclude that you have not contracted out of the Acts.

*Maternity Fee.*

(156) I was engaged to attend a lady with her first baby at £2 2s. weekly in March, and in consequence of this engagement refused another case. The first lady had a miscarriage in the north of England, and did not give me the option of nursing her. Am I entitled to any fee?—*E. B.*

If the miscarriage took place within three months of the date of the expected birth, you are entitled to a fee, which is usually half that which you would have received had your services been required.

*Advice Wanted.*

(157) I am a trained nurse with a three-year certificate and good testimonials from doctor and committee. I have held good appointments, the last for five years as district nurse under a nonconformist committee. Since that time, now a year and a-half ago, I have advertised ten times in The Hospital and answered over three hundred advertisements without success. Lately I have offered my services without salary for the winter months, but with no results.—*A. F.*

It is a most unusal case. Do you think that the fault lies in the wording of your answers?

*Nurses' Home.*

(158) Will you kindly tell me of a home in London where nurses with midwifery training only are taken?—*A.*

Possibly you may find a private home that would take you, but the larger nurses' associations require general training. A midwifery training does not make a "trained nurse." See advertisements.

*Patient.*

(159) Will you kindly tell me the best means of obtaining a patient, as advertising is expensive?—*A. H.*

If you do not advertise, your only alternative is to interest your local medical men and friends in your home, and to ask them to recommend it.

*Massage.*

(160) I am desirous of obtaining a certificate in massage; will you kindly advise me as to the best hospital and the fees?—*M. P.*

The National Hospital for the Paralysed and Epileptic, Queen's Square, Bloomsbury, W.C. Apply to the Secretary for particulars

1. Would you kindly tell me where I could get the best training in massage? 2. A lady suffering from asthma is desirous of spending the winter in a home or institution, as she cannot afford the expense of going away to lodgings as she has done hitherto.—*M. E. P.*

1. See reply to *M. P.*; 2. The Royal West of England Sanatorium, Weston-super-Mare, makes provision for receiving gentlewomen.

**Standard Books of Reference.**

"The Nursing Profession: How and Where to Train." 2s. net; post free 2s. 4d.
"Burdett's Official Nursing Directory." 3s. net; post free, 3s. 4d.
"Burdett's Hospitals and Charities." 5s.
"The Nurses' Dictionary of Medical Terms." 2s.
"Burdett's Series of Nursing Text-Books." 1s. each.
"A Handbook for Nurses." (Illustrated). 5s.
"Nursing: Its Theory and Practice." New Edition. 3s. 6d.
"Helps in Sickness and to Health." Fifteenth Thousand. 5s.
"The Physiological Feeding of Infants." 1s.
"The Physiological Nursery Chart." 1s.; post free, 1s. 3d.
"Hospital Expenditure: The Commissariat." 2s. 6d.
All these are published by the Scientific Press, Ltd., and may be obtained through any bookseller or direct from the publishers, 28 and 29 Southampton Street, London, W.C.

### Travel Notes.

BY OUR TRAVELLING CORRESPONDENT.

### XC.—WINTER DAYS IN ROME.

### ARMENIAN MASS AT ST. ANDREA.

DURING my first visit to Rome I witnessed the Armenian High Mass at St. Andrea delle Valle, and it was such a remarkable sight that I made notes at the time of the gorgeous and varied ecclesiastical vestments worn by those who took part in it. It must have occurred, I see by my diary, in the first week of January, but the exact day I have not noted. There was a very long procession whose advent was heralded by very singular nasal chanting. First came two boys carrying large candlesticks, their long, rather shapeless gowns were of a dull art-shade of blue, with a species of yoke which was gold-colour; then came four in blue with scarlet yokes; then two in yellow with white yokes; then six in crimson with gold yokes; then three in coral-pink with green yokes. All these men and boys had large crosses on their backs, of a colour matching their yokes, and a deep band of the same round the bottom of the skirt. The deacon was all in scarlet with a rich stole made of cloth of gold. He walked backwards all the way censing the priest, who was a fine looking old man with a long grey beard, and a kind of crown on his head made of red velvet embroidered with gold and with a cross on the top. His cope was a vision of splendour, entirely composed of cloth of gold with crimson threads interwoven; the fronts were lined with rich green silk, beyond which, again, was a golden shimmer. He carried a gilded crucifix with red ribbons hanging from it. Behind him came more red-gowned and blue-yoked men, carrying long staves, at the top of which were silver circles enclosing each a singular cross, composed of a cherub's head with four wings. After the procession they all stood in a semi-circle in front of the altar, and chanted in a way such as I have never heard before; I can only say it was a sort of nasal howl, and a little resembled Scotch bag-pipes badly played! At intervals the men with the staves shook them and they jingled like horses' bells. It was a most curious and interesting ceremony; the service was conducted in Greek, and the kiss of peace was invested with more than usual solemnity. It always remains in my mind as one of the most curious and gorgeous scenes presented to my insular eyes in the Eternal City, and I counsel all visitors to make a point of seeing a similar mass if possible. It is not one of the regular and accepted tourists' sights of Rome, and I should probably have missed it but for the kindness of the Consul.

### THE OPERA IN ROME.

It is cheap and good; for 5 lire (about 4s.) you get excellent places and hear and see well. The buying of tickets is quaint: instead of our sumptuous box offices, all enamel and guilding, you generally buy them in the streets at little stalls or counters. The music is very good and the voices of a rare quality, but the mounting of the pieces leaves something to be desired, and that is probably why the opera is so much cheaper abroad; we spend such untold sums in scenery, machinery, etc. . . . that tickets are priced to meet this heavy expenditure. I saw "Faust" in Rome, and the music was beautiful and the acting good, but appearances were not much considered. Margherita, with the voice of an angel, was distinctly stout and of dark complexion, but rouged and whitened to the proper tint for her very palpable wig. Her apotheosis was the funniest thing imaginable; she had just fallen dead, and Faust and Mephistopheles were standing over her, when the back of the scene opened and showed another Margherita, not quite so stout, wobbling up to Heaven on a very shaky pedestal. Mephistopheles, too, was cheated of his prey, for instead of dragging Faus down a fiery trap-door, he went off alone and undramatically at the side. But never mind these little drawbacks; you will greatly enjoy the cheap and really good opera in Italy.

### SHOPPING IN ROME.

I should like to give you a word of warning on this subject. If money is no object (a state of things rarely met with) where you shop is of no consequence, and you cannot do better than follow the always reliable information of Baedeker or Mr. Hare in your choice of shops, but the places mentioned by them are the best and very often extremely dear. To do the thing cheaply you must go as it were into the byeways of the city, and if possible be accompanied by an Italian friend. The natives alone understand the art of bargaining and he must indeed be a poor negotiator who cannot bring down the coveted article at least one-third. The Italian shopmen naturally look upon an English customer as lawful prey, and quite openly at once add on to the prices of his goods. This being firmly pointed out by the aggrieved purchaser, the smiling proprietor is quite ready after due bargaining and compulsion, to climb down, and accept considerably less. They are so pleasant and kindly all the time that one feels no righteous indignation, and the negotiation is conducted with the utmost suavity on both sides. Eschew the well-known shops patronised by the tourists and discover for yourselves small shops in side streets, where the owners are not quite so ready to swallow the succulent foreigner at a gulp. Shopping is sometimes attended with unexpected excitement, due to the tindery temper of the Italians, but they are always far too courteous to show this personally to the unsuspecting stranger. One day at a large and fashionable bookseller's whilst waiting for my parcel to be made up I heard "a few words" between two of the men—presently came the word "stupido," whereupon the younger man dashed a parcel he had in his hand on the floor, flew at number two and smacked his face. Number two took number one by the throat and after a scuffle both fell on the floor. Up they got and waltzed about the shop cuffing and smacking one another whenever and wherever they could get in a blow. Then a third man appeared on the scene (hitherto we had been alone with the combatants) and addressed them entreatingly as "Allessandro!" "Paolo!" they took no notice, but continued to belabour each other. On the arrival of a fourth, peace was restored and we left Allessandro and Paolo amicably engaged in doing up a parcel together—the storm was over—the sun shone again, and the placable Italian nature was once more in the ascendant.

### TRAVEL NOTES AND QUERIES.

BAY OF ST. MALO (Querist).—It is not nearly so cheap as it once was, because it has become so popular. There is nothing now that I know of to come within your terms. If you can come to 6 or 7 francs per day let me know and I will give you addresses. St. Servan is much cheaper than Dinard, but it is not so pretty or airy. St. Enogat too is rather cheaper, but your terms will not secure you anything in the Bay, I fear. If you are not compelled to remain by the sea coast, I think you would do better at Dinace, some 12 miles inland. It is a charming old-world town with an English colony and accommodation is reasonable.

BOOKS ON ROME (Museum).—These are endless. First let us consider fiction: "Daniella," by George Sand; "Ariadne," Ouida; "Tranformation," Hawthorne; "Mlle. Mori," Roberts; "Rome," Zola. Of more solid books: "Roma Sotteranea," Northcote; "Rome, Ancient and Modern," Donovan; "Topography of Rome and its Vicinity," Gill; "The Cities and Cemeteries of Etruria," Dennis. For books neither heavy nor light but most useful and interesting, "Walks in Rome," and "Days near Rome," Augustus Hare; "Roba di Roma," Story, and "Roman Gossip," by Mrs. Elliott.

